# Predictors of Weight Change in Male HIV-Positive Injection Drug Users Initiating Antiretroviral Therapy in Hanoi, Vietnam

**DOI:** 10.1155/2011/890308

**Published:** 2011-07-06

**Authors:** Alice M. Tang, Heidi B. Sheehan, Michael R. Jordan, Dang Van Duong, Norma Terrin, Kimberly Dong, Trinh Thi Minh Lien, Nguyen Vu Trung, Christine A. Wanke, Nguyen Duc Hien

**Affiliations:** ^1^Department of Public Health and Community Medicine, Tufts University School of Medicine, Jaharis 265, Boston, MA 02111, USA; ^2^Department of Medicine, Tufts University School of Medicine, Boston, MA 02111, USA; ^3^Bach Mai Hospital, Center of Pathology, Hanoi, Vietnam; ^4^National Hospital of Tropical Diseases, Hanoi, Vietnam

## Abstract

We examined clinical and nutritional predictors of weight change over two consecutive 6-month intervals among 99 HIV-positive male injection drug users initiating antiretroviral therapy (ART) in Hanoi, Vietnam. The average weight gain was 3.1 ± 4.8 kg in the first six months after ART and 0.8 ± 3.0 kg in the following six months. Predictors of weight change differed by interval. In the first interval, CD4 < 200 cells/*μ*L, excellent/very good adherence to ART, bothersome nausea, and liquid supplement use were all associated with positive weight changes. Moderate to heavy alcohol use and tobacco smoking were associated with negative weight changes. In the second interval, having a CD4 count <200 cells/*μ*L at the beginning of the interval and tobacco smoking were the only significant predictors and both were associated with negative weight changes. We identified several potential areas for interventions to promote weight gain immediately after starting ART in this population. Studies are needed to determine whether improving weight prior to, or at, ART initiation will result in improved outcomes on ART.

## 1. Introduction

Access to antiretroviral treatment (ART) has expanded rapidly in many moderate-to low-income countries affected by the HIV epidemic. In addition to reducing mortality rates, ART has many favorable effects among people living with HIV (PLHIV), such as improving weight and lean body mass, particularly in patients with greater pretreatment immunological and virological compromise [1, 2]. Several large-scale ART programs in sub-Saharan Africa indicate that malnutrition (low BMI) at the start of ART is significantly and independently associated with subsequent mortality [3–6], while weight gain after ART is associated with survival [7, 8]. It is unclear whether this association is causal. Although weight changes appear to parallel the success of ART, it is unknown whether interventions to improve weight prior to or at ART initiation will improve subsequent outcomes. 

In Vietnam, the number of PLHIV is estimated to be 293,000 with an HIV prevalence rate of 0.53% among adults [9]. Injection drug use (IDU) remains the main driver of HIV in Vietnam. The HIV prevalence rate among IDUs is estimated to be 30% overall, with rates of over 50% in Ho Chi Minh City and Quang Ninh [10]. In Hanoi, the most recent estimates of HIV among IDUs are around 25% [9]. Rapid scale-up of ART in Vietnam began in 2005 with support from the Vietnam Ministry of Health, the United States President's Emergency Plan for AIDS Relief (PEPFAR), and the Global Fund. To our knowledge, no studies are published on the nutritional outcomes of ART initiation in Vietnam. 

The primary objective of this analysis was to determine predictors of weight change six to 12 months after initiation of ART among IDUs in Hanoi. Our results will help develop and determine the appropriate timing of targeted nutritional interventions in Vietnam, paving the way for future trials to test the impact of improving weight on ART outcomes.

## 2. Methods

### 2.1. Study Population

The HIV/AIDS outpatient clinic at the National Hospital of Tropical Diseases (NHTD) in Hanoi, Vietnam is a PEPFAR-supported clinic providing ART to approximately 800 HIV-infected patients. Between August 2006 and December 2008, 100 HIV-positive, ART-naïve patients were recruited from the outpatient clinic at NHTD into an ongoing longitudinal study on the causes and consequences of malnutrition in HIV-infection. Patients were eligible if they were HIV seropositive, between the ages of 18 and 65, had a history of IDU within the previous five years, were eligible to start ART, understood the study procedures, and signed informed consent. Since there were few female drug users in the clinic population at the time of recruitment, the study population was restricted to men only. Study participants are followed every 6 months for 3 years. For the current analysis, we include data from the baseline, 6 month, and 12 month study visits. One participant was excluded from this analysis because of missing questionnaire data at baseline. 

This study was reviewed and approved by the Institutional Review Boards of the Tufts School of Medicine and the Hanoi School of Public Health.

### 2.2. Data Collection

Data collected at each study visit included a brief physical examination, body composition measurements, dietary intake, and a lifestyle questionnaire. The lifestyle questionnaire elicited information on sociodemographics; medical history; alcohol, tobacco, and drug use; use of ART and other prescribed medications; adherence to ART; food insecurity. Adherence to ART was assessed by the patient's subjective rating of how well he was able to take all of his prescribed HIV medications in the past 30 days using a 5-point Likert scale with responses of excellent, very good, good, fair, or poor [11]. Food insecurity was measured using a modified version of the USDA's short form of the household food security scale [12]. Dietary intake was estimated by a 24-hour recall. Anthropometric assessments included weight, height, and skinfold measurements (triceps, suprailiac, and subscapular). Fat mass was calculated using the equations of Durnin and Womersley [13]. Fat-free mass (FFM) was obtained by subtracting fat mass from total body weight. Dietary intake and anthropometric assessments were administered by study personnel who were trained and standardized regularly by a research dietitian. At each study visit, blood was collected for the determination of complete blood count, CD4 cell count, and HIV viral load. 

### 2.3. Statistical Analysis

Using a repeated measures regression model, we identified clinical and nutritional predictors of weight change over two consecutive 6-month intervals (Interval 1: pre-ART to 6 months post-ART; and Interval 2: 6 to 12 months post-ART). The unit of analysis was person-intervals. The term “interval baseline” is defined as the baseline (pre-ART) visit for Interval 1 and the 6 month visit for Interval 2. The outcome of interest was weight change over the interval. 

We examined several time-varying predictors of weight change including CD4 count (cells/*μ*L), log viral load (copies/ml), drug use in the previous 6 months (yes/no), hepatitis C coinfection (yes/no), TB coinfection (yes/no), energy intake (total kilocalories from food and supplements), carbohydrate intake (grams), fiber intake (grams and grams/kg body weight), protein intake (grams and grams/kg body weight), total fat intake (grams), food insecurity (yes/no), adherence to ART (Excellent/Very Good versus Good/Fair/Poor), intake of liquid supplements (yes/no), and general symptoms of illness (thrush, mouth sores, nausea, vomiting, diarrhea, stomach pain, and fever). Liquid supplement use was defined as any report of intake of Ensure liquid supplements, Oresol (oral glucose-electrolyte solution), glucose, or sweetened condensed milk which was not part of a meal or other drink. Symptoms of illness were categorized as “yes” only if participants reported that they were bothered by the symptom moderately, quite a bit, or extremely. Moderate/heavy drinking was categorized as “yes” when participants drank ≥4 days per week and/or had 3 or more drinks on the days they drank. All of these variables were measured at the interval baseline, with the exception of adherence to ART, which was taken at the end of the interval since patients were reporting their adherence levels during the previous 6 months. Tobacco smoking (yes/no) was assessed at the baseline visit only. In addition, changes in CD4+ cell count and log viral load over the interval were examined as potential correlates of weight change, while baseline (pre-ART) body mass index (BMI; weight (in kg)/height (in m^2^)) was examined as a potential confounder.

All statistical analyses were carried out using the SAS statistical software (SAS Institute, Cary, NC, Version 9.2). Repeated measures analyses were performed using PROC MIXED in SAS. To determine if the predictors of weight change differed by interval, interaction terms of each potential predictor with an indicator variable for “interval” were examined in the models. Since several of these interaction terms were significant in the final model, for ease of interpretation, the model results are presented separately by interval in Table 3 with standard errors and *P*-values obtained from the Estimate statement in PROC MIXED. 

## 3. Results

Table 1 shows the sociodemographic and clinical characteristics for the 99 men that were assessed at the baseline study visit only. The average age was 32 years. The majority (69%) were married and had 10 or more years of education (79%). Approximately one-third had been imprisoned in their lifetime. Nearly 80% reported current tobacco smoking and almost all of the men (92%) tested positive for Hepatitis C infection. All participants were started on ART approximately 2 weeks after study entry. Most (59%) were started on ZDV/3TC/EFV. For this analysis, we did not take into account any treatment changes or interruptions over the 12 month followup period, which were very few. 

By one year after starting ART, 8 men had died (7 in the first 6 months), 10 men were lost to follow-up, 3 were jailed, and 4 had transferred to another clinic. Thus, of the 100 patients enrolled, 81 and 75 had data available for analysis from their 6 and 12 month follow-up visits, respectively.  Table 2 shows participant characteristics that were measured at each of the three study visits. Nearly half (47%) reported using any drugs (injection or noninjection) and 24% reported injection drug use in the six months prior to recruitment. Rates decreased over time for both types of drug use. While rates of moderate/heavy alcohol use decreased slightly, rates of light drinking increased substantially over time. There were very few reports of food insecurity at any of the study visits. Both CD4 counts and viral load improved over time. Body mass index (BMI) also improved over time. At baseline, BMI was less than 18.5 kg/m^2^ for 39% of our study participants, compared with 23.5% among men of the same age group living in the region [14]. By 12 months post-ART, only 17% of the men had BMI levels <18.5 kg/m^2^. Reports of general symptoms of illness decreased from pre- to post-ART, while energy intake remained constant. The proportion adhering well to ART decreased from 6 to 12 months post-ART.

### 3.1. Treatment Outcomes at 6 and 12 Months after ART Initiation

Table 3 shows the average change for several continuous variables over each interval. Both immunological and virological responses were favorable. The mean increase in CD4 counts was 66 cells/*μ*L in Interval 1 and 32 cells/*μ*L in Interval 2, resulting in mean CD4 levels of 160 cells/*μ*L (median = 137 cells/*μ*L) after six months on treatment and 195 cells/*μ*L (median = 171 cells/*μ*L) after 12 months. However, at the end of 12 months on treatment, 61% (46/75) still had CD4 counts <200, 19% had CD4 <100 (14/75), and 3% (2/75) had CD4 < 50 cells/*μ*L (data not shown). HIV viral load levels decreased by nearly three logs in the first six months and remained stable over the next 6 months. By 12 months of treatment, 92% had viral load levels <1000 copies/mL and 77% had levels <50 copies/mL. 

Average weight and BMI increased significantly over both intervals, but the increases were more substantial in the initial 6 months after starting ART. The increase in weight was approximately equally divided between increases in fat and fat-free mass. Percent fat increased significantly only in the first 6 months.

### 3.2. Predictors of Weight Change

Many of the potential predictors we examined were associated with weight change in the unadjusted regression models (data not shown). In the final multivariate model, several predictors remained significantly and independently associated with weight change. These variables are listed in Table 4. In cases where “interval” was considered a significant effect modifier (all predictors except for tobacco smoking), coefficients are presented and interpreted separately for Interval 1 and Interval 2 as described in Methods. In Interval 1, all of these predictors were significantly associated with a positive weight change, except for moderate/heavy drinking, which was significantly associated with negative weight change. In Interval 2, however, none of these covariates were associated with weight change, except for CD4 < 200 at the interval baseline, which was now significantly associated with negative weight change. Tobacco smoking had the same negative effect on weight change in both intervals (*β* = −1.2 kg; *P* =.02). Energy intake and food insecurity were not independently associated with weight change in either interval. 

**Figure 1:**
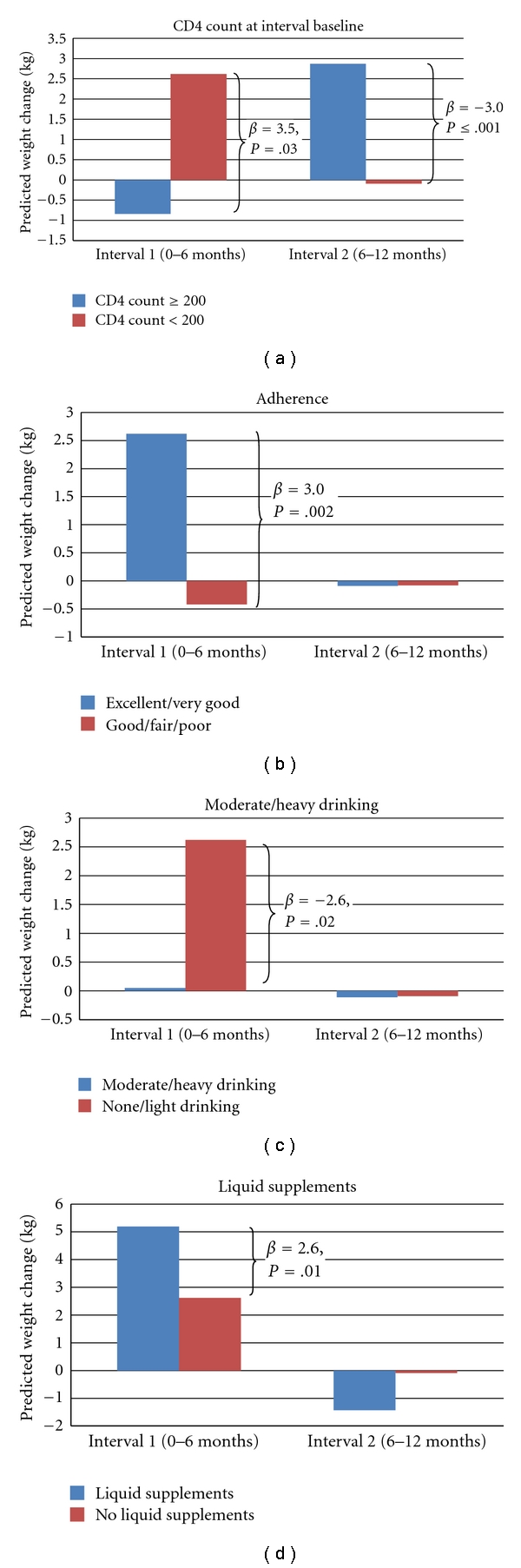
Predicted weight changes by interval for men with and without specific characteristics. (a) Values for other covariates are: Adherence = Excellent/Very Good, Nausea = no, Moderate/Heavy Drinking = no, Liquid supplements = no, and Tobacco smoking = yes. (b) Values for other covariates are: CD4 < 200 = yes, Nausea = no, Mod/Heavy Drinking = no, Liquid supplements=no, and Tobacco smoking = yes. (c) Values for other covariates are: CD4 < 200 = yes, Adherence = Excellent/Very good, Nausea = no, and Liquid supplements = no, and Tobacco smoking = yes. (d) Values for other covariates are CD4 < 200 = yes, Adherence = Excellent/Very good, Nausea = no, and Moderate/Heavy drinking = no, and Tobacco smoking = yes.

Figures 1(a)–1(d) shows the net weight gain/loss by interval for presence and absence of several of the covariates as predicted by the final regression model. For each figure, the values assumed for the other covariates in the model are delineated in the figure title.  Figure 1(a) shows that participants with CD4 < 200 at the start of ART are predicted to gain, on average, over 2.6 kg in the first 6 months of treatment, while those with CD4 ≥200 are predicted to lose over 0.8 kg during that same interval. This difference of nearly 3.5 kg is statistically significant. In Interval 2, the reverse is seen with significantly larger weight gain among those with CD4 counts ≥200.  Figure 1(b) shows the predicted net weight changes by interval for those with Excellent/Very Good adherence to ART compared to those with Good/Fair/Poor adherence. In the first 6 months of ART, those with Excellent/Very Good adherence are predicted to gain 2.6 kg weight, while those reporting Good/Fair/Poor adherence are predicted to lose 0.4 kg. In the second interval, only very slight weight losses are predicted for both levels of adherence. In Figure 1(c), we see that moderate to heavy alcohol intake has a significant negative effect on weight gain in the first six months after initiation of ART with none/light drinkers gaining 2.6 kg of weight, while moderate/heavy drinkers gained only 0.1 kg. There was, however, no difference in weight change by alcohol intake 6 to 12 months later. Conversely, use of liquid supplements had a significant positive effect on weight gain in Interval 1 (people taking liquid supplements gained 5.2 kg versus 2.6 kg weight gain in all others), but not in Interval 2 (Figure 1(d)).

## 4. Discussion

Overall, ART outcomes were encouraging in this population of Vietnamese men with a history of IDU. CD4 counts increased by 66 cells/*μ*L after six months and by 98 cells/*μ*L after 12 months. HIV viral load decreased significantly, resulting in 92% of the men being virally suppressed (<1000 copies/ml) after 12 months on therapy. Significant increases in weight were observed, particularly in the first 6 months of therapy. 

The pattern of weight gain we observed appears to be consistent with previous reports, with the highest rate of weight gain occurring in the first six months after ART initiation, then stabilizing afterwards. In a combined analysis of patients in ART programs in Cambodia (*n* = 2451) and Kenya (*n* = 2618), Madec et al. [8] observed a similar pattern of weight gain, although patients in that study continued to gain weight up to 12 months post-ART before weights stabilized. The amount of weight gain we observed over the initial six months (3.1 ± 4.8 kg) is also similar to reports from other populations. In 488 patients initiating ART in four African countries (Ethiopia, Kenya, Rwanda, and Uganda), an average weight gain of 3.9 ± 5.1 kg over 6 months was recorded [15]. In India, investigators reported an average weight gain of 2.8 ± 5.4 kg over 6 months in 190 patients starting ART [16]. In 185 Nigerian patients followed up for two years, the average weight of the group increased from 52 kg pre-ART to 59 kg post-ART [17]. In comparison, the average weight in our cohort increased from 53 kg pre-ART to 57 kg after one year post-ART. 

We also observed some differences in our population compared to previous publications. In terms of BMI, Barth et al. [18] reported an average BMI increase of 2.4 kg/m^2^ after 6 months on ART and 3.5 kg/m^2^ after 12 months among patients initiating ART in South Africa. We observed an average BMI increase of only 1.1 kg/m^2^ after 6 months and 1.4 kg/m^2^ after 12 months post-ART. One reason for this difference could be that BMI at baseline was slightly higher (less room to improve) in our participants (median = 19.2 kg/m^2^) compared to the male participants in the Barth study (median = 18.6 kg/m^2^); however patients in the Barth study achieved a BMI level of 23.4 kg/m^2^ after 12 months on ART, whereas our participants achieved a BMI level of only 20.8 kg/m^2^ after 12 months. Another reason is that the Barth study focused on a population where the primary mode of HIV transmission is heterosexual, whereas ours was a population of injection drug users where other related risk factors (behavioral and/or biological) could inhibit optimal weight gain. In addition, although two previous studies reported that patients with lower BMI at ART initiation (≤16 or ≤17) had larger weight gains than those with higher BMI [8, 19], baseline BMI was not associated with weight change in our population. 

We found several significant predictors of weight gain, particularly in the first six months after ART initiation. Patients with more advanced HIV infection at baseline (CD4 cell counts <200 cells/*μ*L) were more likely to have positive weight changes in the first six months of therapy, likely due to the beneficial effects of ART. However, six months after start of ART, patients with CD4 < 200 cells/*μ*L (a sign of continued immunosuppression) had significantly less weight change over the following six months compared to those with CD4 ≥ 200. The vast majority (92%) of our patients started ART with CD4 counts <200 cells/*μ*L. Of these, 87% of patients whose CD4 counts improved to ≥200 cells/*μ*L after 6 months of therapy gained weight, while only 48% of those with continued immunosuppression after 6 months gained weight. 

In our cohort, “Excellent” to “Very Good” adherence to ART medications was significantly associated with positive weight change in the first six months of therapy. Similarly, Ross-Degnan et al. found that adherence was significantly associated with weight gain over the first 9 months after ART initiation in four African countries [15]. In our study, after six months of therapy, excellent/very good adherence was no longer independently associated with positive weight change after taking into account the positive effect of CD4 counts rising to ≥200 cells/*μ*L.

We found that bothersome nausea at baseline (reported by 49% of participants) was associated with weight gain in the first 6 months of ART. While this may seem to be counterintuitive, we can speculate that having bothersome nausea at baseline (pre-ART) is a symptom of illness contributing to reduced food intake, and when these patients initiate ART their symptoms resolve and they are able to eat more and gain more weight. In the second interval, the 15% who reported bothersome nausea (post-ART) had a negative weight change (although not statistically significant (*P* =  .13)). At this point in time, continued nausea or nausea from side effects of the medications may inhibit a patients' ability to gain weight.

The use of liquid supplements was a significant predictor of weight gain in the first six months of ART and therefore may have potential as a nutritional intervention for weight gain (or reducing weight loss) in this population. No other dietary factors were associated with weight change, indicating that a dietary supplement may be necessary. Liquid supplements were primarily Ensure or Oresol (a glucose-electrolyte solution). While Ensure is known as a balanced nutritional supplement, Oresol is generally used as a treatment for dehydration or diarrhea and has little nutritional value beyond its sugar and electrolyte content. The other two types of liquid supplements that were reported in this population (glucose solution and sweetened condensed milk) contribute high amounts of calories from carbohydrates and sugars assisting in weight gain, but have minimal nutrient content. More in-depth information is needed before planning a nutritional intervention with a liquid supplement, such as the reasons why patients were taking these supplements, how much and how often they were taking them, and whether they were taking them on their own or as prescribed by a doctor. Fundamentally, we still need to understand whether weight gain by any means is associated with improved outcomes on ART, or if nutritionally balanced supplements are more likely to be successful. We were not able to examine this in our population because we did not have enough patients taking each of the different types of supplements. 

Another interesting finding from our study is that alcohol intake was associated with negative weight gain in the first six months after ART initiation in the first interval, but not the second. While nutritional status in alcoholics has been described in the United States and Western Europe [20–24], the nutritional impact of alcohol abuse has not been studied in a population with marginal nutritional status such as our population of drug users with HIV in Vietnam. In addition, the effects of alcohol abuse on the liver are more marked in persons with chronic viral hepatitis [25–27]. This is of particular concern in our population where 92% are coinfected with Hepatitis C. Twenty percent of our population reported moderate to heavy amounts of alcohol use at baseline and this group had significantly less weight gain than non- to light drinkers. Further research is needed on the specific nutritional, biological, and/or behavioral effects of alcohol use in this population to determine the mechanism through which it reduces weight change. Similarly tobacco smoking had a significantly negative effect on weight change in both intervals. Since the vast majority (~80%) of our study population reported tobacco smoking at baseline, this would be another high-priority area for research, including the testing of smoking cessation interventions. 

Our study had some limitations. First, our results may not be generalizable to populations where injection drug use is not the major mode of HIV transmission. Injection drug users may have additional complications predisposing to weight loss, such as cytokine-mediated weight loss due to sepsis and infections and/or psychiatric comorbidities (e.g., depression, euphoria, behavioral abnormalities, and memory disturbances) that may affect food choices and eating patterns. Second, this study did not take into account the effect of other medical comorbidities potentially associated with weight change, such as opportunistic infections, inflammatory states, cancers, and malabsorption. We were not able to obtain standardized clinical diagnoses of medical co-morbidities in this study as diagnostic tests for these conditions are not generally available to this patient population and the physicians do not routinely look for these during their ART visits. However, we did include self-reports of TB and *Penicillium marneffei* diagnoses on the questionnaire, but found very few participants who reported these. Finally, a single 24-hour dietary recall to assess dietary intake is a limitation as this may not accurately reflect typical daily intake over the previous 6 or 12 months. We felt that this method for assessment of dietary intake was best suited for this population in Vietnam as it is less of a burden to study participants compared to the food diaries, and we were not aware of a validated food frequency questionnaire in Vietnam. We did ask participants whether the 24-hour recall was reflective of their usual intake, considerably more, or considerably less than their usual intake and 70–80% of participants at 6 and 12 months reported that it was reflective of their usual intake.

In summary, we found that significant weight gain was achieved in the first 6 months after ART initiation, and then stabilized over the next 6 months. While this is a positive result, some improvements are still needed. Although 39% were classified as underweight at baseline, only 28% of our participants gained at least 10% of their baseline weight (an indicator of treatment success used by some ART clinics) by six months after ART initiation, and only one additional person reached this endpoint by 12 months. More research is needed to determine why weight gain did not continue after 6 months of therapy, particularly when a significant proportion of patients in this population were still underweight (BMI < 18.5). In addition, the magnitude of CD4 change over 6 and 12 months of ART use was less than that reported in other populations and this requires further investigation. The fact that having a CD4 < 200 cells/*µ*L at 6 months post-ART was associated with continued weight loss (or a reduction in weight gain) in the following six months suggests that efforts should be made to diagnose and treat patients earlier, before their CD4 levels drop too far below 200 cells/*µ*L that they remain under this threshold after six months of therapy.

This study is the first step towards developing targeted interventions to improve nutritional and immunological outcomes on ART in drug users in Vietnam. We identified several potential areas for interventions to promote weight gain in this population. Studies are still needed to determine whether improving BMI prior to ART initiation will reduce early mortality rates. 

## Figures and Tables

**Table 1:** Characteristics measured at baseline study visit only for 99 HIV-positive men recruited from the National Hospital of Tropical Diseases (NHTD) in Hanoi, Vietnam.

Baseline characteristic	Mean ± SD or N(%)
Age	31.7 ± 4.8
Marital Status^1^	
Never married	22 (22%)
Married	68 (69%)
Divorced/Sep/Widowed	8 (8%)
Education	
<9 years	21 (21%)
10–12 years	43 (43%)
Vocational	18 (18%)
University or more	17 (17%)
Jail or prison (ever)	31 (31%)
Tobacco smoking^1^	77 (79%)
Hepatitis C	91 (92%)
Initial ART Regimen^2^	
ZDV/3TC/EFV	48 (59%)
ZDV/3TC/NVP	19 (23%)
D4T/3TC/NVP	9 (11%)
D4T/3TC/EFV	4 (5%)
DDI/ABC/KAL	1 (1%)

Abbreviations: ZDV: zidovudine; 3TC, lamivudine; EFV: efavirenz; NVP: Nevirapine; D4T: stavudine; ABC: abacavir; KAL: kaletra.

^1^ *n* = 98.

^2^ *n* = 81.

**Table 2:** Characteristics measured at each study visit for 99 HIV-positive men recruited from the National Hospital of Tropical Diseases (NHTD) in Hanoi, Vietnam.

	Study visit (Mean ± SD or N (%))		
	Baseline (pre-ART) (*n* = 99)	6 month (*n* = 81)	12 month (*n* = 75)
Any drug use in last 6 months^1^	47 (47%)	26 (33%)	22 (30%)
Injection drug use in last 6 months^1^	24 (24%)	10 (13%)	8 (11%)
Alcohol^2^			
Nondrinker	52 (53%)	30 (38%)	23 (31%)
Light drinker	26 (27%)	36 (45%)	40 (54%)
Moderate or heavy drinker	20 (20%)	14 (18%)	11 (15%)
Food insecurity^2^	5 (5%)	2 (3%)	3 (4%)
CD4 count (cells/*μ*L)	96.7 ± 67.6	160.3 ± 114.7	194.6 ± 144.5
CD4 <200 cells/uL	88 (89%)	61 (75%)	46 (61%)
Log viral load (copies/mL)^3^	4.9 ± 1.0	2.1 ± 1.0	1.9 ± 0.8
Viral load <1000 copies (%)^3^	7 (7%)	70 (86%)	67 (92%)
Weight (kg)	52.9 ± 7.1	56.5 ± 7.8	57.3 ± 8.0
BMI (kg/m^2^)	19.1 ± 2.1	20.4 ± 2.3	20.8 ±2.3
BMI categories			
< 17.0 kg/m^2^	15 (15%)	4 (5%)	0 (0%)
17.0 to <18.5 kg/m^2^	24 (24%)	15 (19%)	13 (17%)
18.5 to <20 kg/m^2^	26 (26%)	22 (27%)	16 (21%)
≥ 20.0 kg/m^2^	34 (34%)	40 (49%)	46 (61%)
Bothersome nausea^1^	46 (46%)	11 (14%)	15 (20%)
Bothersome Diarrhea^1^	20 (20%)	1 (1%)	1 (1%)
Bothersome fever^1^	62 (63%)	12 (15%)	13 (18%)
Energy intake (kcals/day)^4^	2108 ± 755	2015 ± 799	2192 ± 780
Excellent/Very Good Adherence to ART^5^	—	60 (76%)	51 (69%)

^1^ *n* = 80 for 6 month visit and *n* = 74 for 12 month visit.

^2^ *n* = 98 for baseline, *n* = 80 for 6 month visit, and *n* = 74 for 12 month visit.

^3^ *n* = 73 for 12 month visit.

^4^ *n* = 69 for 12 month visit.

^5^ *n* = 79 for 6 month visit and 74 for 12 month visit.

**Table 3:** Average changes in selected variables over each six month interval.

	Mean ± SD (*P*-value)	
	Interval 1:	Interval 2:
	pre-ART to 6 months post-ART	6 to 12 months post-ART
	(*N* = 81)	(*N* = 75)

CD4 count (cells/*μ*L)	65.6 ± 97.2 (<0.001)	32.4 ± 101.0 (0.01)
Log Viral Load (copies/mL)	−2.8 ± 1.5 (<0.001)	−0.1 ± 0.9 (0.28)
Weight (kg)	3.1 ± 4.8 (<0.001)	0.8 ± 3.0 (0.02)
% Weight change	6.2 ± 9.6 (<0.001)	1.3 ± 5.5 (0.01)
BMI (kg/m^2^)	1.1 ± 1.7 (<0.001)	0.3 ± 1.1 (0.05)
Fat-free mass (kg)	1.5 ± 3.0 (<0.001)	0.5 ± 2.3 (0.05)
Fat-free mass (%)	−1.7 ± 4.0 (<0.001)	−0.3 ± 3.2 (0.48)
Fat mass (kg)	1.6 ± 2.9 (<0.001)	0.3 ± 2.3 (0.27)
Fat mass (%)	1.7 ± 4.0 (<0.001)	0.3 ± 3.2 (0.48)

**Table 4:** Coefficients derived from final multivariate model predicting weight change, accounting for effect modification by Interval.

	Difference in weight change (*β*) ± SE (*P*-value)		*P*-value for interaction term
	Interval 1	Interval 2	
Intercept	−2.7 ± 1.8 (.14)	4.1 ± 0.9 (<.001)	0.001
CD4 <200 cells/*μ*L (yes/no)	3.5 ±1.5 (.03)	−3.0 ± 0.7 (<.001)	<0.001
Adherence to HIV meds (Excellent/Very Good versus Good/Fair/Poor)	3.0 ± 0.9 (.002)	−0.01 ± 0.7 (.98)	0.01
Nausea (yes/no)	2.2 ± 0.8 (.006)	−1.3 ± 0.8 (.13)	0.005
Moderate/Heavy Drinker (yes/no)	−2.6 ± 1.0 (.02)	−0.02 ± 0.8 (.98)	0.06
Liquid supplements (yes/no)	2.6 ± 1.0 (.01)	−1.3 ± 1.5 (.39)	0.05
Tobacco smoking	−1.2 ± 0.5 (.02)		0.17

Interaction term (Tobacco smoking ∗ Interval) was dropped from the final model due to nonsignificant *P*-value. Coefficient shown is from main effect of tobacco smoking only.
